# Structural factors associated with SARS-CoV-2 infection risk in an urban slum setting in Salvador, Brazil: A cross-sectional survey

**DOI:** 10.1371/journal.pmed.1004093

**Published:** 2022-09-08

**Authors:** Mariam O. Fofana, Nivison Nery, Juan P. Aguilar Ticona, Emilia M. M. de Andrade Belitardo, Renato Victoriano, Rôsangela O. Anjos, Moyra M. Portilho, Mayara C. de Santana, Laiara L. dos Santos, Daiana de Oliveira, Jaqueline S. Cruz, M. Catherine Muenker, Ricardo Khouri, Elsio A. Wunder, Matt D. T. Hitchings, Olatunji Johnson, Mitermayer G. Reis, Guilherme S. Ribeiro, Derek A. T. Cummings, Federico Costa, Albert I. Ko

**Affiliations:** 1 Department of Epidemiology of Microbial Diseases, Yale School of Public Health, New Haven, Connecticut, United States of America; 2 Instituto de Saúde Coletiva, Universidade Federal da Bahia, Salvador, Bahia, Brazil; 3 Instituto Gonçalo Moniz, Fundação Oswaldo Cruz, Salvador, Bahia, Brazil; 4 Department of Biostatistics, University of Florida, Gainesville, Florida, United States of America; 5 Department of Mathematics, University of Manchester, Manchester, United Kingdom; 6 Faculdade de Medicina da Bahia, Universidade Federal da Bahia, Salvador, Bahia, Brazil; 7 Department of Biology, University of Florida, Gainesville, Florida, United States of America; 8 Emerging Pathogens Institute, University of Florida, Gainesville, Florida, United States of America; Harvard University, UNITED STATES

## Abstract

**Background:**

The structural environment of urban slums, including physical, demographic, and socioeconomic attributes, renders inhabitants more vulnerable to Severe Acute Respiratory Syndrome Coronavirus 2 (SARS-CoV-2) infection. Yet, little is known about the specific determinants that contribute to high transmission within these communities. We therefore aimed to investigate SARS-CoV-2 seroprevalence in an urban slum in Brazil.

**Methods and findings:**

We performed a cross-sectional serosurvey of an established cohort of 2,041 urban slum residents from the city of Salvador, Brazil between November 2020 and February 2021, following the first Coronavirus Disease 2019 (COVID-19) pandemic wave in the country and during the onset of the second wave. The median age in this population was 29 years (interquartile range [IQR] 16 to 44); most participants reported their ethnicity as Black (51.5%) or Brown (41.7%), and 58.5% were female. The median size of participating households was 3 (IQR 2 to 4), with a median daily per capita income of 2.32 (IQR 0.33–5.15) US Dollars.

The main outcome measure was presence of IgG against the SARS-CoV-2 spike protein. We implemented multilevel models with random intercepts for each household to estimate seroprevalence and associated risk factors, adjusting for the sensitivity and specificity of the assay, and the age and gender distribution of our study population. We identified high seroprevalence (47.9%, 95% confidence interval [CI] 44.2% to 52.1%), particularly among female residents (50.3% [95% CI 46.3% to 54.8%] versus 44.6% [95% CI 40.1% to 49.4%] among male residents, *p* < 0.01) and among children (54.4% [95% CI 49.6% to 59.3%] versus 45.4% [95% CI 41.5% to 49.7%] among adults, *p* < 0.01). Adults residing in households with children were more likely to be seropositive (48.6% [95% CI 44.8% to 52.3%] versus 40.7% [95% CI 37.2% to 44.3%], *p* < 0.01). Women who were unemployed and living below the poverty threshold (daily per capita household income <$1.25) were more likely to be seropositive compared to men with the same employment and income status (53.9% [95% CI 47.0% to 60.6%] versus 32.9% [95% CI 23.2% to 44.3%], *p* < 0.01). Participation in the study was voluntary, which may limit the generalizability of our findings.

**Conclusions:**

Prior to the peak of the second wave of the COVID-19 pandemic, cumulative incidence as assessed by serology approached 50% in a Brazilian urban slum population. In contrast to observations from industrialized countries, SARS-CoV-2 incidence was highest among children, as well as women living in extreme poverty. These findings emphasize the need for targeted interventions that provide safe environments for children and mitigate the structural risks posed by crowding and poverty for the most vulnerable residents of urban slum communities.

## Abstract 2

Mariam O. Fofana and colleagues investigate SARS-CoV-2 seroprevalence in an urban slum in Brazil.

## Author summary

**Why was this study done?:**

- Inhabitants of informal settlements or urban slums are at increased risk of Severe Acute Respiratory Syndrome Coronavirus 2 (SARS-CoV-2) infection during the ongoing pandemic, yet there is limited evidence on the heterogeneities in risk experienced by populations in these high-transmission settings.
- Understanding the factors that influence risk within slum populations can inform public health prevention strategies in marginalized and under-resourced communities.
- We wanted to identify sociodemographic factors, such as age and gender, that influence SARS-CoV-2 infection risk among residents of urban slum communities.

**What did the researchers do and find?:**

- We collected sociodemographic data and measured levels of SARS-CoV-2 anti-S IgG in serum samples from 2,041 urban slum residents from Salvador, Brazil after the first pandemic wave, from November 2020 to February 2021.
- Overall, 48% of residents had positive antibody levels, reflecting prior SARS-CoV-2 infection.
- We found that women and children were more likely to have experienced a SARS-CoV-2 infection.

**What do these findings mean?:**

- Residents of this urban slum community experienced a high risk of SARS-CoV-2 infection during the initial wave of the epidemic in Brazil.
- Even within the high-risk environment of urban slums, there are superimposed gradients of risk that make women and children particularly vulnerable to Coronavirus Disease 2019 (COVID-19).

## Introduction

More than 1 billion people who reside in urban slums or informal settlements are at increased risk of Coronavirus Disease 2019 (COVID-19) and also most likely to suffer loss of employment and income due to disease control measures such as lockdowns [1,2]. Although several studies have demonstrated an association of socioeconomic deprivation with increased COVID-19 risk and mortality, such studies have generally relied on ecological designs or have compared poor communities to wealthier ones [3,4]. Moreover, studies relying on passive reporting of cases may suffer from surveillance bias. Prior studies of transmissible and non-transmissible diseases have shown variations in risk not only between communities but also within socially deprived environments, where certain segments of the population may be particularly vulnerable [5,6]. Yet, there remains little known about the Severe Acute Respiratory Syndrome Coronavirus 2 (SARS-CoV-2) risk gradients within urban slum communities and how these risk gradients may inform targeted interventions for this vulnerable population. Although the conditions and structure of each slum community are unique, there may be valuable common insights: regardless of geography, slums are invariably densely populated, have poor infrastructure, lack of access to services, and are inhabited by residents who experience significant housing and financial insecurity.

Brazil is among the countries that have suffered the highest burden of COVID-19, with nearly 680,000 deaths reported as of August 2022 among its 210 million inhabitants over the course of several epidemic waves, the first of which occurred between May and September 2020 [7]. Brazil also has roughly 28 million people—15% of its urban population—residing in slum settlements within cities [1]. Prior to the pandemic, we had been performing long-term longitudinal follow-up of a cohort from the city of Salvador, Brazil to characterize the transmission dynamics and burden of leptospirosis [8], dengue [9], chikungunya [10], and Zika [11] among urban slum residents. Herein, we describe the findings of a serological survey of this cohort conducted after the first epidemic wave in Brazil in 2020, which aimed to examine the socioeconomic and structural factors associated with SARS-CoV-2 infection in the urban slum setting and characterize the gradients of risk within these deprived communities.

## Methods

### Study site and population

**Fig 1:**
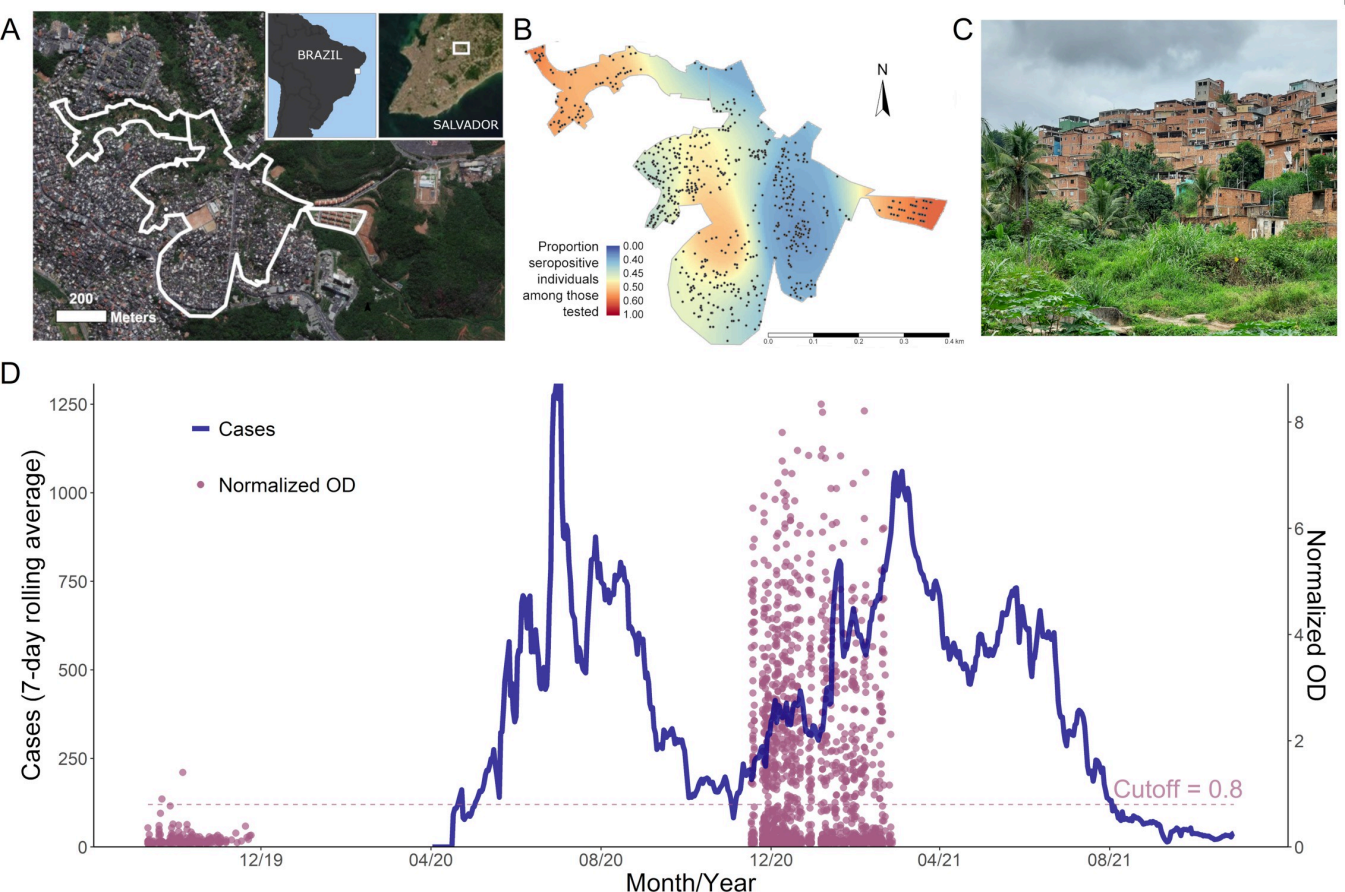
Study population and context. Panel (A) shows an aerial image of the study area, with insets depicting the location of the study area within Brazil and Salvador. Panel (B) depicts the location of participating households. The choropleth reflects the spatial distribution of seropositivity within the study area. Panel (C) is a representative photo of the study area. (D) Confirmed cases of COVID-19 in Salvador, Brazil, up to October 2021 (blue). Overlaid over the epidemiologic curve, in purple, are the normalized OD values of serological samples collected from cohort participants prior to the pandemic (September 11 to November 24, 2019) and after the first wave of the pandemic (November 17, 2020 to February 26, 2021). The map in panel (A) was created using ArcGIS software by ESRI. Source: ESRI, Maxar, GeoEye, Earthstar Geographics, CNES/Airbus DS, USDA, USGS, AeroGRID, IGN, HERE, Garmin, OpenStreetMap contributors, and the GIS User Community. Base layers: https://services.arcgisonline.com/ArcGIS/rest/services/World_Imagery/MapServer, https://services.arcgisonline.com/arcgis/rest/services/Canvas/World_Dark_Gray_Base/MapServer. COVID-19, Coronavirus Disease 2019; OD, optical density.

Our study was conducted in the Pau da Lima community in Salvador, Brazil. Geographically, Pau da Lima consists of hills and valleys, with a population of approximately 25,000 inhabitants per the most recent national census [12]. The study site, shown in Fig 1A–1C, encompasses 4 valleys, covering a densely populated area of 0.35 km^2^. Approximately half of the households do not hold legal titles to their homes, and more than 70% of the heads of household earn less than the Brazilian minimum wage [13]. Since 2002, a collaborative research team has maintained a longitudinal open cohort of residents in this community, which has been previously described [8,9,11,14,15]. Study teams canvas households located within the study area and perform a census (number of residents, age, and gender of residents) in households where at least 1 adult resident is present at the time of the study visit and is able to provide such information. Individuals who meet eligibility criteria (sleeping 3 nights or more per week within the study area, aged 2 years or more) and who provide written informed consent (parental consent for minors <18 years of age) are recruited for participation in household and serological surveys, which have been conducted annually or semiannually since 2002. The most recent serosurvey prior to the COVID-19 pandemic was conducted from September to November 2019 (Fig 1D). For the present study, we conducted a household census as described above and recruited a cross-sectional panel of participants to measure SARS-CoV-2 seropositivity (details in S1 Appendix, “Study recruitment”). Individuals who consented to participate had a similar age distribution, but a higher proportion of female participants compared to individuals who declined (Table A in S1 Appendix).

### Data collection

Surveys and serum samples were collected between November 18, 2020 and February 26, 2021, after the first epidemic wave and in the beginning of the second wave (Fig 1D). The study period preceded the rollout of vaccines to the general population in February 2021, such that the presence of SARS-CoV-2 antibodies was reflective of prior infection. A community field research team conducted a census of each household, listing the number of residents, gender, and number of people per room. The head of household contributed information on the income of each wage-earning resident in the household. Household income per capita was estimated as the total income of all residents divided by the number of residents in each household. Participants who consented also completed a detailed individual-level survey, including data on COVID-19 exposures, COVID-19 cases in the household, preventive behaviors (e.g., handwashing, mask use), medical history, and symptom history. The survey includes participant-reported socioeconomic variables such as type of employment (formal versus informal), race/ethnicity, and level of education. Survey data were recorded and managed using REDCap software hosted at the Instituto Gonçalo Moniz (Fiocruz Bahia) [16]. Blood samples were collected by venipuncture from consenting participants and stored in a cooler for transport to the laboratory facility. After centrifugation, the obtained sera were aliquoted and maintained at −20°C until analysis.

### Laboratory methods

We measured levels of immunoglobulin G against the SARS-CoV-2 spike protein (anti-S IgG) in serum samples collected from all cohort participants after the first epidemic wave and in stored serum samples collected during a prior survey before the introduction of SARS-CoV-2 in Brazil. Anti-S IgG levels were measured using a commercially available ELISA kit (Euroimmun AG, Lübeck, Germany), which has a sensitivity of 91.1% (95% CI 80.7% to 96.1%) and specificity of 100% (95% CI 96.5% to 100%) [17]. Following the manufacturer’s recommendations, samples were diluted 1:100 in sample buffer, and a set of calibrator, positive, and negative control samples were included on each plate. Optical density (OD) was measured at a wavelength of 450 nm, and normalized values were derived by dividing the OD value of each test sample by the value of the calibrator. All samples with a normalized OD ≥ 0.8 were defined as positive, based on an estimation of optimal cutoffs (Fig B in S1 Appendix) using a previously described Bayesian mixture model [18]. We consider a higher cutoff (normalized OD ≥ 1.1) in sensitivity analyses.

### Data analysis

Initial analyses were planned in February 2021, after completion of data collection. The primary outcome was SARS-CoV-2 seropositivity as assessed by anti-S IgG. We visualized the relationship between age and seroprevalence using a generalized additive model considering age as a continuous variable. We adapted a previously described multilevel Bayesian logistic model incorporating household-level clustering [19] to derive estimates of seroprevalence that are adjusted for the sensitivity and specificity of the assay and the age and gender distribution of our study population. Independent variables in regression models included demographic, socioeconomic, and household variables as assessed from the household and individual surveys. We assessed measures of association using univariable logistic regression. Variables with a statistically significant (*p* < 0.05) association with SARS-CoV-2 seropositivity in a multivariable logistic regression model were retained in our final models.

As our initial descriptive analyses revealed higher seroprevalence among participants aged <18 years compared to adults, and among female participants, we conducted additional analyses with stratification by age and gender, as well as household composition, employment, and income, to elucidate the underlying factors. We performed a multilevel, multivariable logistic regression that incorporated a random intercept at the household level to estimate the effects of age on SARS-CoV-2 seropositivity in our study population. In addition to the main analysis comparing children to adults, we conducted preplanned analyses further categorizing participants <13 and 13 to 17 years of age. To elucidate whether children contribute to higher transmission, we estimated the effect of presence of children in the household on SARS-CoV-2 seropositivity among adults. We assessed model fit based on Akaike information criterion (AIC) to select the most parsimonious explanatory model.

We hypothesized that the effect of presence of children may be mediated by household size, and we estimated this indirect effect using mediation models. We fitted a Poisson model to predict number of residents per household and a binomial model for seropositivity, incorporating age, gender, and household clustering. We estimated the direct and indirect effects and bootstrap confidence intervals using the R “mediation” package [20]. We explored associations between gender and sociodemographic characteristics (income, employment) that are potential confounders of the observed association between gender and SARS-CoV-2 seropositivity.

Participants with missing values for independent variables were excluded from the relevant analyses (pairwise deletion). There were no missing values for the variables included in our final regression models. We conducted sensitivity analyses using data from households in which we obtained sera from all participants, to assess the robustness of our findings to missing serology data. We conducted a final set of analyses to derive seroprevalence estimates adjusted for assay performance. We applied post-stratification of age- and gender-specific estimates to compare seroprevalence estimates between study participants and residents who declined participation.

All analyses were conducted using the software R, version 4.1.3 [21]. Analysis code is available at: https://github.com/m-fofana/SalvadorSARSCoV2. This study is reported as per the Strengthening the Reporting of Observational Studies in Epidemiology (STROBE) guidelines (S1 STROBE Checklist).

### Ethical considerations

The study was approved by the Institutional Review Boards of the Instituto Gonçalo Moniz, Oswaldo Cruz Foundation (Fiocruz) and the Brazilian National Commission for Ethics in Research (CAAE 35405320.0.1001.5030 and 17963519.0.0000.0040), and the Yale University Human Research Protection Program (2000031554).

## Results

### Population demographics

A total of 2,041 individuals (1,460 adults and 581 children) in 951 households were recruited among 2,681 eligible residents (Fig A in S1 Appendix). Similar to previous surveys, we observed higher participation among female residents. In 431 households, we obtained serological samples from every resident of the households (979 individuals). Table 1 describes the demographic and socioeconomic characteristics of the study population. The median age was 29 years (interquartile range [IQR] 16 to 44) and 58.5% were female. The median size of participating households was 3 (IQR 2 to 4), with a median household daily per capita income of 2.32 (IQR 0.33 to 5.15) US Dollars (USD). Most participants reported their ethnicity as Black (51.5%) or Brown (41.7%). Overall, 35.2% (503/1,431) of adult participants had 6 or fewer years of formal education, and 51.4% (751/1,460) were unemployed. Most participants reported frequent (every day or most days) adherence to non-pharmaceutical interventions such as handwashing (70.5%), alcohol-based hand sanitizer use (67.0%), and face mask use (72.4%); in contrast, fewer participants reported frequent isolation (43.4%) or physical distancing of ≥2 meters in public spaces (46.6%).

**Table 1:** Study population and demographic characteristics.

Variable (N)	Category	Number (%)	IgG +	Raw seroprevalence (95% CI)
**All participants**				
**Age**	<18	581 (28.5%)	333	57.3 (53.2%–61.4%)
(2,041)	18–29	450 (22.0%)	222	49.3 (44.6%–54.0%)
	30–44	516 (25.3%)	227	44.0 (39.0%–48.0%)
	45–59	332 (16.3%)	130	39.2 (33.9%–44.7%)
	≥60	162 (7.9%)	70	43.2 (35.5%–51.2%)
**Gender**	Female	1193 (58.5%)	598	50.1 (47.3%–53.0%)
(2,041)	Male	848 (41.5%)	384	45.3 (41.9%–48.0%)
Race	Black	1042 (51.5%)	481	46.2 (43.1%–49.2%)
(2,024)	Brown	844 (41.7%)	422	50.0 (46.0%–53.0%)
	Other	38 (1.9%)	20	52.6 (36.1%–68.7%)
	White	100 (4.9%)	50	50.0 (40.0%–59.0%)
**Daily per capita income**	<1.25	760 (49.8%)	396	52.1 (48.5%–55.7%)
(USD) (1,525)	1.25–2.49	235 (15.4%)	101	43.0 (36.0%–49.0%)
	2.5–4.99	212 (13.9%)	84	39.6 (33.1%–46.6%)
	≥5	318 (20.9%)	144	45.3 (39.8%–50.9%)
**Adults**				
**Years of schooling**	0–6	503 (35.2%)	210	41.7 (37.4%–46.2%)
(1,431)	7–9	296 (20.7%)	142	48.0 (42.0%–53.0%)
	>9	632 (44.2%)	287	45.4 (41.5%–49.4%)
Marriage or stable union	No	952 (65.2%)	433	45.5 (42.3%–48.7%)
(1,460)	Yes	508 (34.8%)	216	42.5 (38.2%–47.0%)
Employment	Formal	201 (13.8%)	92	45.8 (38.8%–52.9%)
(1,460)	Informal	339 (23.2%)	138	40.7 (35.5%–46.2%)
	Unspecified	169 (11.6%)	75	44.4 (36.8%–52.2%)
	Unemployed	751 (51.4%)	344	45.8 (42.2%–49.4%)
Individual income	<1.25	60 (6.0%)	34	56.7 (43.3%–69.0%)
(999)	1.25–2.49	107 (10.7%)	44	41.1 (31.8%–51.1%)
	2.5–4.99	169 (16.9%)	74	43.8 (36.2%–51.6%)
	≥5	663 (66.4%)	288	43.4 (39.6%–47.3%)

Employment, education, and marriage were assessed for adult (≥18 years) participants. Variables with statistically significant effects in univariable analyses are indicated in bold.

CI, confidence interval; USD, US Dollar.

### Seroprevalence

Fig 1D shows the serology assay results for the cohort, overlaid on the epidemic curve of confirmed COVID-19 cases in Salvador, Brazil. Among 195 samples that were selected and tested from the pre-pandemic survey (from September 9 to November 11, 2019), 2 (1.0%) were positive. Fitting the observed anti-S IgG levels with a Bayesian mixture model revealed little overlap between the predicted distributions of OD values for individuals with and without a serological response (Fig B in S1 Appendix). Based on this model, a cutoff of 0.8 has greater than 95% specificity in identifying serological response (Table B in S1 Appendix).

Of the 2,041 samples collected from November 2020 to February 2021, a total of 982 (48.1%, 95% confidence interval [CI] 45.9% to 50.3%) had positive IgG levels against SARS-CoV-2. The estimated seroprevalence adjusted for household-level clustering and test characteristics was 47.9% (95% CI 44.2% to 52.1%) among study participants. The seroprevalence among all eligible residents, as estimated by post-stratification, was 47.7% (95% CI 44.0% to 51.8%) (Table C in S1 Appendix). Among the 982 seropositive individuals, 327 (33.3% [95% CI 30.4% to 36.4%]) reported having at least 1 COVID-19 related symptom (cough, coryza, sore throat, nasal congestion, shortness of breath, fever, chills, anosmia, dysgeusia, myalgia, arthralgia, nausea, vomiting, diarrhea, or headache) since the beginning of the pandemic. The frequency of reported symptoms was lower among seronegative individuals (24.5% [95% CI 21.9% to 27.2%], difference 8.8% [95% CI 4.8% to 12.9%]). The proportion of households with at least 1 seropositive individual was 56.2%. Of the 179 participants who reported known contact with a COVID-19 case within their household, only 1 reported a case resulting in death.

### Risk factors

**Fig 2:**
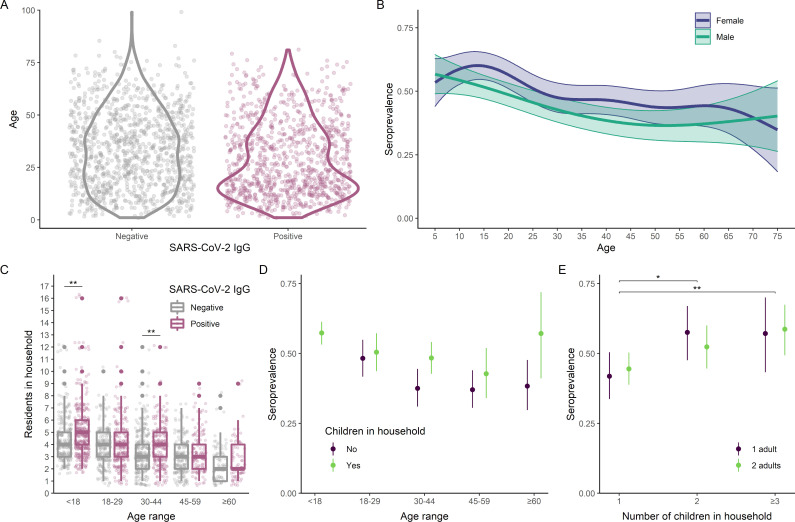
Seroprevalence among children and in their households. (A) Age distribution of SARS-CoV-2 seronegative (median 32 years, IQR 19–46) and seropositive (median 25 years, IQR 15–41) individuals. (B) SARS-CoV-2 seroprevalence and 95% CI associated with age, as estimated with a generalized additive model. (C) Distribution of household size by age among SARS-CoV-2 seronegative and seropositive individuals. Seropositive individuals tended to be in larger households compared to seronegative individuals in the same age group. (D) Seroprevalence and 95% CI stratified by age group and presence of children in the household. (E) Variation in seroprevalence among children by household composition (number of children and number of adults). The number of other children in the household was associated with higher seroprevalence, but there was no statistically significant difference between households with 1 adult and those with 2 adults. Asterisks indicate statistically significant differences (2-tailed *t* test, Bonferroni-adjusted *p* < 0.05: *; <0.01: **; <0.001: ***; α = 0.01, 0.0125, 0.0167 in panels (C–E). CI, confidence interval; IQR, interquartile range; SARS-CoV-2, Severe Acute Respiratory Syndrome Coronavirus 2.

Estimates of seroprevalence adjusted for test characteristics and household-level clustering were significantly higher among children (54.4% [95% CI 49.6% to 59.3%) than adults (45.4% [95% CI 41.5% to 49.7%], *p* < 0.01) and in female (50.3% [95% CI 46.3% to 54.8%]) compared to male (44.6% [95% CI 40.1% to 49.4%], *p* < 0.01) participants (Table 1, Table C in S1 Appendix, and Fig 2A and 2B). Additional variables associated with seropositivity in univariable analyses included low per capita household income and number of residents per household. We did not observe a significant association with race (Table 1 and Table D in S1 Appendix). Reported adherence to non-pharmaceutical interventions was not significantly associated with the risk of seropositivity (Table E in S1 Appendix). Because risk factors (including structural environment of the home, health attitudes, and preventive behaviors) are likely to be correlated among members of a household, we further conducted an analysis using a multilevel multivariable model to account for household-level clustering of risk.

### Household composition

Household size was larger among seropositive individuals (median 4 (IQR 3 to 5) versus 3 (IQR 2 to 4)) (Fig 2C), and women were more likely to be the sole adults in households with multiple children (Fig C in S1 Appendix). Adults residing in households with children were more likely to be seropositive (48.6% [95% CI 44.8% to 52.3%] versus 40.7% [95% CI 37.2% to 44.3%], *p* < 0.01). The effect of the presence of children was more prominent among adults aged 30 to 44 years and those aged 60 years or more (Fig 2D). Children were also more likely to be seropositive if they lived with other children, but there was no significant difference in seroprevalence between children who lived with 1 adult and those who lived with 2 adults (56.7% [95% CI 49.1% to 64.0%] versus 53.2% [95% CI 47.0% to 59.4%], *p* = 0.54, Fig 2E). After adjusting for household clustering, gender, and total number of residents in the household, the OR for SARS-CoV-2 seropositivity among children compared to adults aged 30 to 44 was 2.07 [95% CI 1.42 to 3.03] (Table 2, Model 1).

**Table 2:** Effect (odds ratio) of age and presence of children in the household.

Variable	Category	OR (95% CI)		
		*Model 1*	*Model 2*	*Model 3*
Age	<18	**2.07 (1.42–3.03)**		
	18–29	**1.57 (1.07–2.30)**	**1.62 (1.16–2.26)**	**1.53 (1.10–2.13)**
	30–44	(Ref)	(Ref)	(Ref)
	45–59	0.88 (0.58–1.36)	0.97 (0.67–1.41)	0.97 (0.67–1.41)
	≥60	0.95 (0.53–1.70)	0.96 (0.58–1.58)	0.99 (0.60–1.62)
Gender	Male	(Ref)	(Ref)	(Ref)
	Female	**1.49 (1.13–1.96)**	**1.51 (1.13–2.03)**	**1.49 (1.11–1.99)**
Daily per capita income		1.03 (0.98–1.07)	1.02 (0.98–1.07)	1.03 (0.99–1.07)
(USD)				
Children in home (Y/N)			1.32 (0.98–1.78)	
Total residents		**1.14 (1.04–1.25)**		**1.13 (1.05–1.23)**
*AIC*		*2*,*001*	*1*,*476*	*1*,*470*
*ICC*		*0*.*328*	*0*.*139*	*0*.*129*

Variables included in each model are: age category, gender, and household daily per capita income (Model 1, Model 2, and Model 3); total number of residents in the household (Model 1 and Model 3); and presence of children in the household (Model 2). Models 2 and 3 include only adults. Effects with statistically significant confidence intervals are indicated in bold.

AIC, Akaike information criterion; CI, confidence interval; ICC, Intraclass correlation coefficient; OR, odds ratio.

To examine whether the higher seroprevalence observed among adults living with children was mediated by increased crowding in such households, we implemented binomial regression models with random intercepts per household to account for clustering, and we compared several alternative models. First, we compared a baseline model including a binary variable for the presence of children in the household (Table 2, Model 2) to an alternative model including the total number of residents in the household (Table 2, Model 3). Model 3 demonstrated a better fit based on the AIC. A mediation analysis to test whether the effect of presence of children is mediated by the total number of residents showed a statistically significant indirect effect (*p* < 0.01), accounting for an estimated 80% of the total effect (Table 3).

**Table 3:** Mediation analysis for the effect of presence of children in the household.

	Coefficient	Bootstrap 95% CI	*p*-Value
Average indirect effect	0.051	0.019–0.083	<0.01
Average direct effect	0.012	−0.051–0.076	0.73
Total effect	0.063	0.006–0.119	0.03
Proportion mediated	0.800	0.187–4.112	0.03
*Independent variables (individual level)*: *Age*, *gender*			
*Independent variable (household level)*: *Presence of children*			
*Mediator*: *Total residents in household*			
*Random effects*: *Random intercept by household*			
*Outcome*: *SARS-CoV-2 seropositivity*			

The analysis was performed by estimating a Poisson regression model for the hypothesized mediator (number of residents in the household) and a binomial regression model for the outcome (SARS CoV-2 anti-S IgG positivity). Both the mediator model and the outcome model include age, gender, and presence of children in the household as independent variables. The outcome model additionally includes the number of residents in the household as an independent variable.

CI, confidence interval; SARS-CoV-2, Severe Acute Respiratory Syndrome Coronavirus 2.

### Poverty and unemployment among women

**Fig 3:**
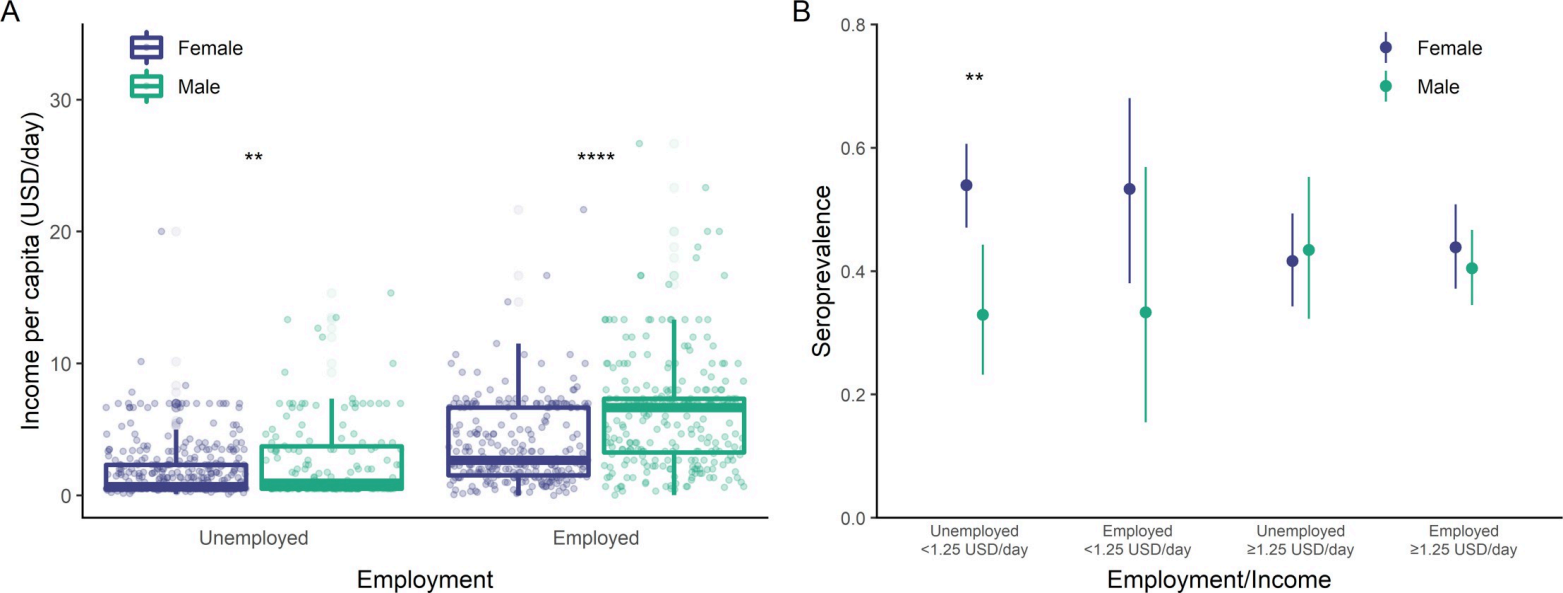
Socioeconomic vulnerability among women. (A) Household income (per capita) by employment status and gender. (B) Seroprevalence by household income (per capita), employment status, and gender. Asterisks indicate statistically significant differences (2-tailed *t* test, Bonferroni-adjusted *p* < 0.05: *; <0.01: **; <0.001: ***; α = 0.0125 in panels (A and B)). USD, US Dollar.

Women were more likely to be unemployed than men (61.7% [95% CI 58.4% to 64.9%] versus 35.5% [95% CI 31.6% to 39.6%]) and had lower income per capita in their households (Fig 3A) regardless of employment status (median [IQR] 2.67 [1.53 to 6.67] versus 6.67 [3.25 to 7.33] USD/day among employed adults; 0.67 [0 to 2.32] versus 0.94 [0 to 3.73] USD/day among unemployed adults). After accounting for income and employment, seroprevalence remained higher among women compared to men (Fig 3B). Women who were unemployed and had a household per capita income below 1.25 USD/day were significantly more likely to be seropositive compared to men with the same employment and income status (Fig 3B).

### Sensitivity analyses

In additional analyses considering data only from households in which all residents provided a serum sample (Figs D–F in S1 Appendix), using a higher cutoff for seropositivity (Table F in S1 Appendix), adding additional age strata (<13 and 13 to 17; Table G in S1 Appendix), and using multiple imputation to account for missing data (Table H in S1 Appendix), our findings of higher seroprevalence among children and women, and in households with children, remained unchanged.

## Discussion

From the early days of the COVID-19 pandemic, there was recognition that the fragile structural and social environment of urban slums constitutes an important yet poorly understood risk [2,22]. Although studies have described socioeconomic disparities in COVID-19 risk and outcomes [23,24], few have focused on the particular risk of urban slums. In this community-based study conducted in the city of Salvador, Brazil, we found that nearly half of residents in an urban slum had evidence of SARS-CoV-2 infection after the first wave of the epidemic in 2020 and before the peak of the second wave. In contrast, cross-sectional surveys conducted among blood donors in the states of São Paulo and Rio Grande do Sul estimated 10% to 30% seroprevalence in February 2021, when our data collection ended [25,26]. This finding is consistent with studies in other settings that reported higher seroprevalence in slums than in non-slum settings [4,27], highlighting the increased vulnerability of socioeconomically deprived communities.

We were able to collect detailed demographic and socioeconomic data at both individual and household levels and overcome the limitations of previous studies that investigated COVID-19 risk in slum compared to non-slum areas, but did not examine the gradients of risk within a slum community [27,28]. Our findings suggest that even within an overall socioeconomically deprived environment, there is a gradient of risk associated with sociodemographic characteristics and household composition.

We found that children in this urban slum setting had high SARS-CoV-2 seroprevalence, significantly greater than observed in adults, which sharply contrasts with patterns of transmission in high-income countries and urban populations within these countries. Most studies to date have reported lower or similar seroprevalence of COVID-19 among children compared to adults during the early phase of the pandemic [29]. Serosurveys relying on residual clinical samples have reported higher prevalence among children, but may not be reflective of the general population of children who do not regularly undergo blood draws [30–32]. The community-based design of our study allows for better comparability of seroprevalence between children and adults. Prior studies of respiratory viruses such as influenza showed that school-aged children are reservoirs of transmission [33,34]. We found that adults aged 30 to 44 and 60 years or more were more likely to be SARS-CoV-2 seropositive if they lived with children, suggesting that the multigenerational composition of urban slum households contributes to infection risk in specific adult groups (likely parents and grandparents). However, we were unable due to the cross-sectional design to determine whether children were more likely to be the index case within households.

Although SARS-CoV-2 seropositivity was associated with larger household size, the OR for seropositivity among children remained statistically significant in models that included the total number of household residents, suggesting that the high seropositivity in children cannot be exclusively attributed to household size. Schools in Salvador closed in-person instruction for a prolonged period from March 2020 to May 2021 [35]. Yet, children may have continued to socialize in similarly assortative patterns within their urban slum community (e.g., contact with other children in neighboring houses). The combination of school closures and the lack of safe childcare options may have resulted in higher exposure to SARS-CoV-2 in high-risk urban slum environments, as suggested by the high seroprevalence observed in this study.

We found that women in this urban slum community had significantly higher risk compared to men. Although there is evidence for sex differences in the immune response to SARS-CoV-2 infection and disease severity [36], serological surveys have generally found similar or lower prevalence among women compared to men [37]. Thus, the gender difference in risk observed in our study was likely driven by social rather than biological determinants. Interestingly, higher seroprevalence among women has also been reported in other urban slum settings: two serosurveys conducted in urban slums in Mumbai and Bangalore, India reported a higher seroprevalence among women than men, but did not investigate potential mediators of this effect [4,28]. We found that women who were unemployed and had the lowest household incomes were significantly more likely to be seropositive for SARS-CoV-2. Thus, the disparities between slum and non-slum areas are compounded by additional disparities within slum populations resulting in exacerbated SARS-CoV-2 infection risk for the poorest women.

A limitation of this study is that our data do not allow for identification of time of infection nor of primary cases within households, such that we were unable to identify which household members were first to be infected and estimate what proportion of SARS-CoV-2 transmission occurred within households. Although waning of antibody titers may result in missed identification of individuals with a prior infection, serological surveys offer important alternative and complementary data on community transmission that captures asymptomatic infections with minimal bias from differences in test-seeking and access. Our observational design does not allow for a complete decomposition of the complex interactions between gender, employment, income, household structure, and other unmeasured variables: households with children are larger, and larger households have lower per capita income, such that regression model estimates are not truly independent. Nevertheless, our data highlight the need for targeted interventions (e.g., local, cost-free access to prevention and treatment) that address the risk experienced by the most vulnerable segments of slum communities, such as women who are unemployed, and the poorest households.

Potential sources of bias include selection bias as participants in the study may differ from residents who chose not to participate, although our post-stratified estimates of seroprevalence among all eligible residents were similar to estimates among study participants. Ascertainment and recall bias cannot be excluded, as many of the exposure variables were participant-reported. Although survival bias may result in lower estimates of seroprevalence, mortality was relatively low in Salvador (2.5% in February 2021) [38] and in our study population. We did not have complete serological data for all household residents, but in sensitivity analyses using only data from households with serological results for all residents, our findings remained consistent. While the survey was initiated during a period of lower incidence following the first wave, an unforeseen second wave led to rising incidence during the period of data collection. Our seroprevalence estimates may thus reflect infection risk attributable to both the first and the second waves of transmission. Finally, this study was initiated relatively early in the pandemic. With the emergence of multiple variants of concern causing rapidly successive incidence surges and the deployment of vaccines, more sophisticated methods will be needed to infer epidemic trends from serological surveillance data.

## Conclusions

Our study provides key insights into the gradient of SARS-CoV-2 infection risk within the deprived environment of urban slums, leveraging a large community-based cohort with detailed demographic and socioeconomic data at both individual and household levels. While the specific microenvironment of each community is unique, Pau da Lima shares features with urban slum communities in general: vulnerability to COVID-19 derives from the physical environment (construction quality, crowding, poor ventilation, poor access to sanitation facilities), as well as the social environment (financial precarity, mobility, social contact patterns) [39,40]. For example, slum residents are less able to isolate as they must travel to work and maintain an income and depend on often crowded and poorly ventilated public transportation to places of work typically located far from slum areas [41].

As has become evident, effective responses to the COVID-19 pandemic must include not only biomedical interventions but also the deployment of social support systems to mitigate the profound disruptions caused by the disease itself and control measures [42,43]. Participants in our study reported high adherence to hand hygiene and mask use but less frequent social and physical distancing, possibly because such measures are less feasible and effective in the crowded environment of slums. Moreover, given that the structural factors (poverty, crowding) that we have identified may not be readily modified in the short term, implementing and guaranteeing access to interventions that can mitigate the resultant risk (e.g., vaccination, therapeutic treatment, ventilation in public spaces) is crucial to achieve an equitable pandemic response.

## Supporting information

S1 STROBE Checklist Checklist of items that should be included in reports of cross-sectional studies. (DOC)

S1 Appendix Additional details of methods and sensitivity analyses. Table A in S1 Appendix. Demographic characteristics of eligible residents and study participants. Table B in S1 Appendix. Specificity associated with model-derived seropositivity thresholds. Table C in S1 Appendix. Adjusted seroprevalence estimates. Table D in S1 Appendix. Univariable logistic regression analyses of individual and household characteristics associated with seropositivity. Table E in S1 Appendix. Adherence to non-pharmaceutical interventions. Table F in S1 Appendix. Sensitivity analysis with higher seropositivity threshold. Table G in S1 Appendix. Sensitivity analysis with different age categories. Table H in S1 Appendix. Multivariable analyses with multiple imputation. Fig A in S1 Appendix. Study eligibility and recruitment flowchart. Fig B in S1 Appendix. Observed and fitted distributions of normalized OD values. Fig C in S1 Appendix. Household composition. Fig D in S1 Appendix. Comparison of households with complete serological data and overall study population. Fig E in S1 Appendix. Sensitivity analysis on effect of age among households with complete serological data. Fig F in S1 Appendix. Sensitivity analysis on the effects of gender, income, and household composition among households with complete serological data.
(PDF)

## Abbreviations

AIC: Akaike information criterion
CI: confidence interval
COVID-19: Coronavirus Disease 2019
ICC: intraclass correlation coefficient
IQR: interquartile range
OD: optical density
SARS-CoV-2: Severe Acute Respiratory Syndrome Coronavirus 2
USD: US Dollar
