# Supplementary material for: Structural factors associated with SARS-CoV-2 infection risk in an urban slum setting in Salvador, Brazil: A cross-sectional survey
Source: PLoS Med. 2022 Sep 8;19(9):e1004093. doi: 10.1371/journal.pmed.1004093 (PMC9499230; doi:10.1371/journal.pmed.1004093)
Supplement: S1 Appendix — Table A in S1 Appendix. Demographic characteristics of eligible residents and study participants. Table B in S1 Appendix. Specificity associated with model-derived seropositivity thresholds. Table C in S1 Appendix. Adjusted seroprevalence estimates. Table D in S1 Appendix. Univariable logistic regression analyses of individual and household characteristics associated with seropositivity. Table E in S1 Appendix. Adherence to non-pharmaceutical interventions. Table F in S1 Appendix. Sensitivity analysis with higher seropositivity threshold. Table G in S1 Appendix. Sensitivity analysis with different age categories. Table H in S1 Appendix. Multivariable analyses with multiple imputation. Fig A in S1 Appendix. Study eligibility and recruitment flowchart. Fig B in S1 Appendix. Observed and fitted distributions of normalized OD values. Fig C in S1 Appendix. Household composition. Fig D in S1 Appendix. Comparison of households with complete serological data and overall study population. Fig E in S1 Appendix. Sensitivity analysis on effect of age among households with complete serological data. Fig F in S1 Appendix. Sensitivity analysis on the effects of gender, income, and household composition among households with complete serological data. (PDF) [file pmed.1004093.s002.pdf]

Structural factors associated with SARS-CoV-2 infection risk in an urban slum setting in Salvador, Brazil: A cross-sectional survey

Appendix

Mariam O. Fofana, Nivison Nery Jr, Juan P. Aguilar Ticona, Emilia M.M. de Andrade Belitardo, Renato Victoriano, Rôsangela O. Anjos, Moyra M. Portilho, Mayara C. de Santana, Laiara L. dos Santos, Daiana de Oliveira, Jaqueline S. Cruz, M. Catherine Muenker, Ricardo Khouri, Elsio A. Wunder Jr, Matt D.T. Hitchings, Olatunji Johnson, Mitermayer G. Reis, Guilherme S. Ribeiro, Derek A.T. Cummings, Federico Costa, Albert I. Ko

Study recruitment

The study area is subdivided into four valleys. Because different studies and field teams were active in each valley prior to the onset of the COVID-19 pandemic, there was some variation in the census and recruitment process. In valleys 1, 2, and 4, study teams visited every household in November 2020 to February 2021 to perform a detailed census of dwellings in the study area, including building use (residential, commercial, empty, abandoned, or in construction), geolocation, and the number and demographic characteristics of residents in each household. Household residents who were present at the time of the visit were eligible for participation. The study teams visited households multiple times to maximize recruitment. Sociodemographic surveys were completed in 725 of the 1276 residential households in valleys 1, 2, and 4 (Fig A).

In valley 3, a simple random sample of 300 out of 782 residential households was selected for participation in the present study, and in 163 of these households at least one resident participated in the sociodemographic survey. A total of 377 out of 593 residents in the randomly selected households participated in the sociodemographic survey. Additionally, 229 (out of 300) residents in 89 households that had not been initially selected volunteered to participate. Of these 606 individuals, 516 consented to participate in the serologic survey.

Demographic data were available for 2,480 of the 2,681 eligible residents. Table A shows the age and gender distribution of all eligible residents for whom demographic data were available, including those who declined (N = 433) and those who consented (N = 2047) to participate in the study. All eligible residents who consented to participate underwent an individual survey as well as collection of a serum sample to measure SARS-CoV-2 antibody levels (Figure A). Overall, the study population had a similar age distribution but a higher proportion of female residents compared to eligible residents who did not participate in the study (Table A).

Figure A: Study eligibility and recruitment flowchart.

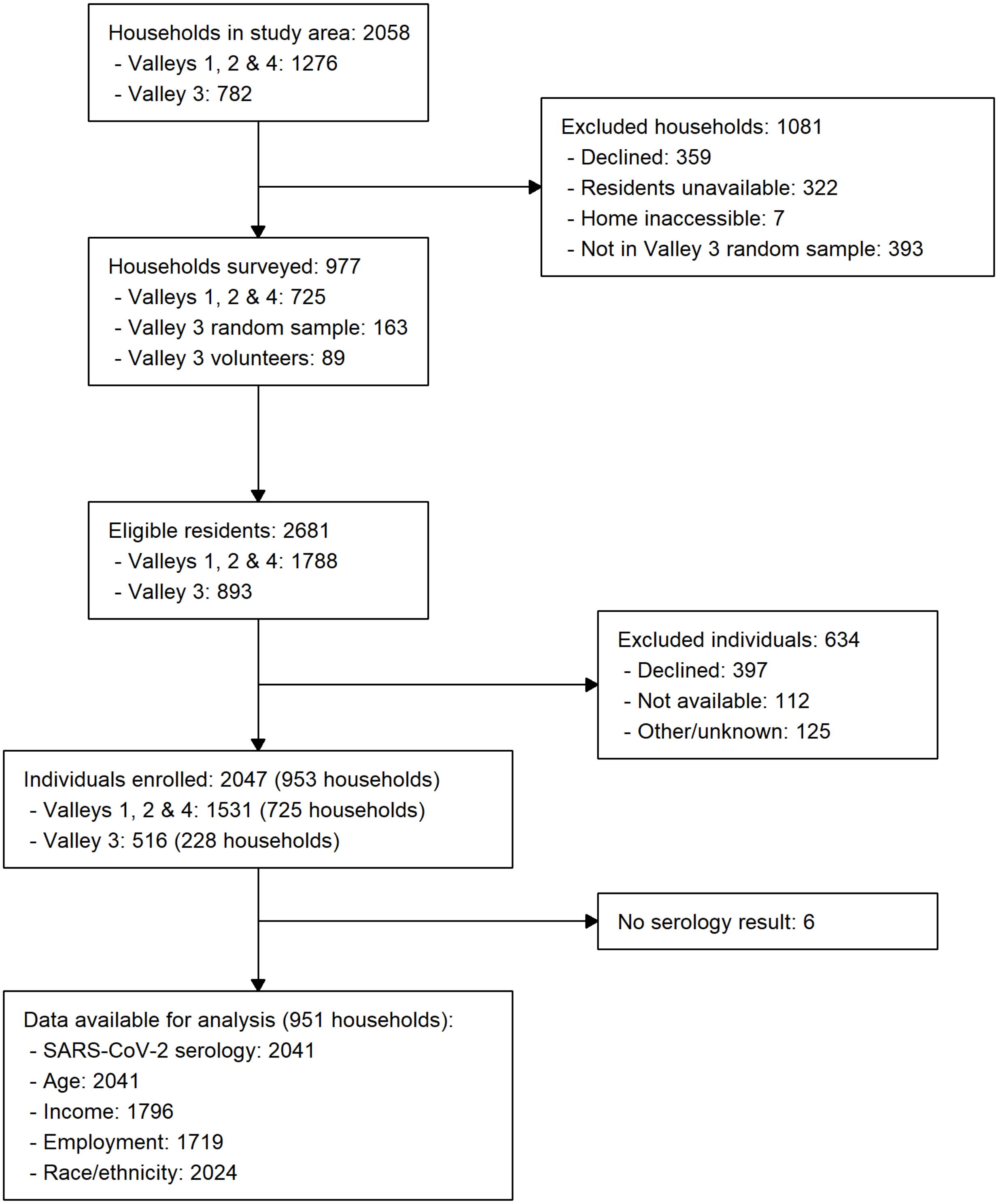

Table A: Demographic characteristics of eligible residents and study participants. Age is shown as median and interquartile range. Percentages are shown with 95% confidence intervals. p-values (Chi-squared test) are shown for comparisons between participants who consented to participate, and participants who declined but for whom demographic data were available.

Variable	All eligible residents (N=2480)	Declined (N=433)	Consented (N=2047)	p-value
**Age**	29 [16-44]	28 [17-43]	29 [16-44]	0.41
**% <18 years old**	28.1% [26.3-29.9%]	25.5% [21.4-30.1%]	28.6% [26.6-30.6%]	0.23
**% Female**	56.6% [54.6-58.6%]	43.6% [37.9-49.5%]	58.5% [56.3-60.6%]	**<0.01**
Gender distribution by age group				
**% Female, age <18**	54.1% [46.6-61.4%]	60.0% [36.4-80.0%]	53.4% [45.4-61.2%]	0.75
**% Female, age 18-29**	60.1% [56.0-64.1%]	44.3% [32.6-56.6%]	62.3% [57.9-66.4%]	**<0.01**
**% Female, age 30-44**	52.5% [48.6-56.5%]	48.9% [33.9-64.0%]	52.8% [48.7-56.9%]	0.72
**% Female, age 45-59**	61.9% [56.8-66.7%]	46.3% [32.8-60.3%]	64.5% [59.0-69.6%]	**0.02**
**% Female, age >=60**	55.7% [51.4-59.9%]	39.1% [29.0-50.2%]	58.9% [54.2-63.4%]	**<0.01**

Distribution of normalized optical density values

The distribution of normalized OD values in our sample suggests that there is overlap in antibody levels between individuals with and without SARS-CoV-2 infection, as observed in prior studies [1,2]. Per the manufacturer, certain test values (≥ 0.8 and < 1.1) are considered borderline, but it is unclear whether such results are more likely to represent infected or non-infected patients. We therefore conducted a sensitivity analysis, considering alternate cutoff values obtained from a Bayesian mixture model, which is an extension of a previously published model by Hitchings et al [3,4].

The model assumes that there are two separate sub-populations: participants with and without SARS-CoV-2 antibodies. Given proposed prior distributions for the OD values in these two sub-populations, we evaluated the likelihood of the observed data and use an MCMC algorithm to yield posterior distributions. We assumed that normalized OD values fit a mixture of two Gaussian distributions. We based the prior distribution for individuals without serological response (non-responders) on the observed values from samples collected prior to the COVID-19 pandemic (mean 0.141, SD 0.143). The prior parameters for normalized OD values among individuals with a measurable serological response were drawn from uniform distributions.

We then implemented an MCMC algorithm with a chain length of 250,000, a burn-in of 125,000 simulations and an acceptance ratio of 0.2. The likelihood was computed as follows:

$$l(\theta) = \sum_i log\left(p * f_R(x_i) + (1-p) * f_{NR}(x_i)\right)$$

where p is the probability of being a responder, $f_R(x)$ is the pdf of the seroresponse distribution, and $f_{NR}(x)$ is the pdf of the non-response distribution.

Panel A in Figure B shows the observed (dashed black line) density of normalized OD values and the posterior distributions obtained from the Bayesian mixture model (non-responders in green, responders in purple, combined distribution in black). Table B shows the specificity associated with various OD thresholds to identify individuals with a serological response. For a specificity of 99.5%, the model-derived cutoff is 1.05, similar to the manufacturer-recommended cutoff of 1.1 (Fig B, panel B). When stratifying by age group, the model-derived cutoff was slightly higher among participants 15 years and younger compared to older participants (Fig B, panel C), but the cutoff remained within the manufacturer-suggested range. In order to confirm that the observed higher seroprevalence among women was not due to differences in antibody response (i.e., women having higher antibody levels and thus being more likely to be identified as seropositive), we compared the distribution of normalized OD values (Fig B, panel D) by gender. A difference in antibody response would be reflected by a shift in the density curves on the x-axis, which we did not observe.

Figure B: Observed and fitted distributions of normalized OD values. Panels A and C show the observed distribution of normalized OD values (dashed black lines) for the overall study population (A) and by age group (C). Fitted distributions obtained from the MCMC mixture model are shown for seropositive (purple line), seronegative (green line), and all (solid black line) individuals. Panel B shows the model-predicted probability of SARS-CoV-2 seropositivity. Panel D shows the distribution of normalized OD values among male and female participants. The gray dashed lines (panels A, B, D) represent the manufacturer-recommended cutoffs of 0.8 and 1.1 for the anti-S1 IgG ELISA.

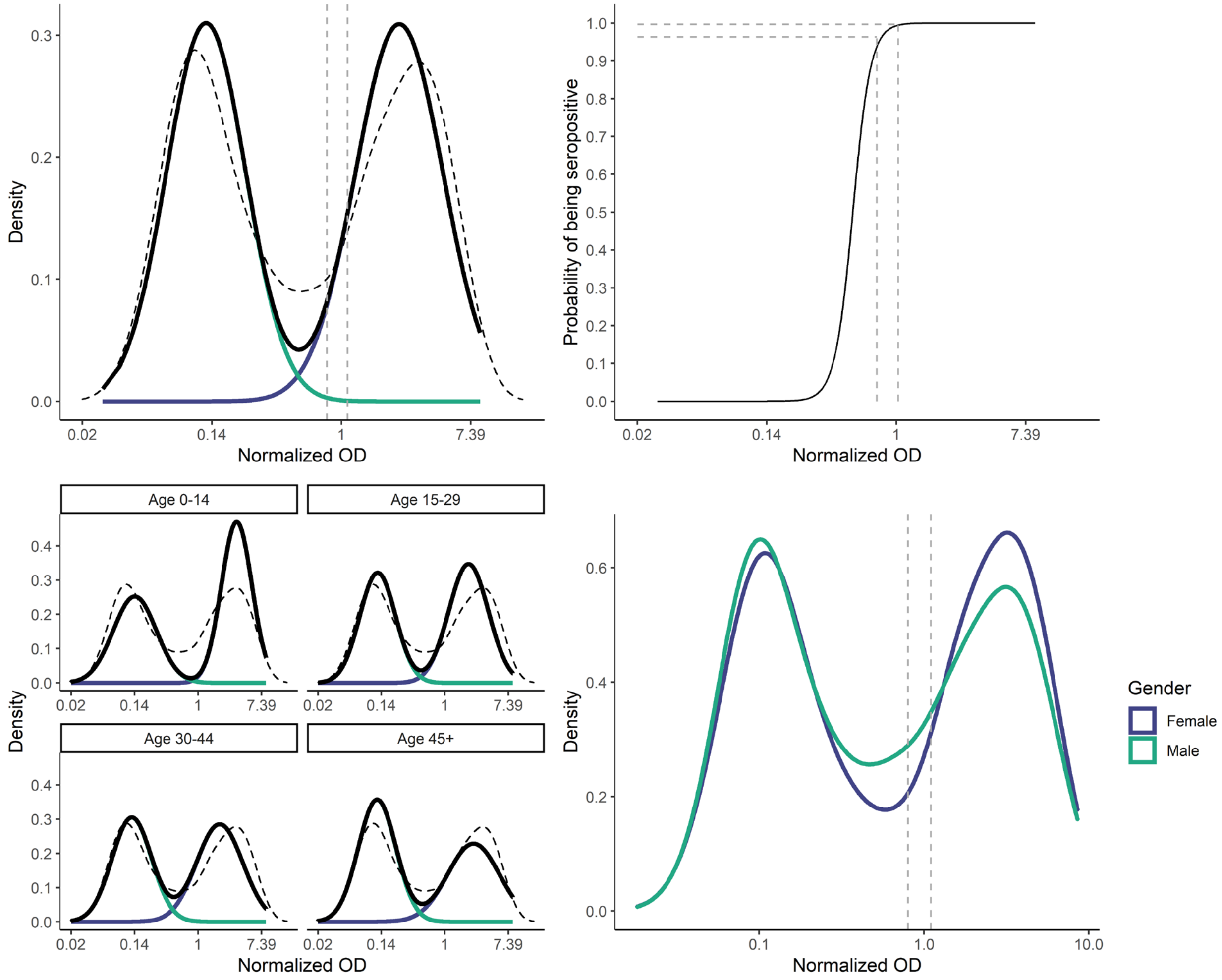

Table B: Specificity associated with model-derived seropositivity thresholds.

Normalized OD threshold	Specificity
0.765	95.0%
0.869	98.0%
0.955	99.0%
1.048	99.5%
1.295	99.9%

Adjusted seroprevalence estimates

We derived seroprevalence estimates that incorporate uncertainty due to imperfect test sensitivity and specificity using a previously described Bayesian hierarchical logistic regression model that also accounts for household-level clustering [5]. Briefly, the model incorporates a random effect for each household, and draws the observed ELISA result for each individual from a binomial distribution based on the manufacturer-reported test performance. In addition to the overall seroprevalence, we derived age- and gender-specific estimates. By applying these estimates to the age and gender distribution of residents in our study area, we derived post-stratified estimates of seroprevalence that account for differences in the demographic makeup of study participants compared to the population of eligible residents (Table C).

Table C: Adjusted seroprevalence estimates. Model-derived estimates are shown for each age and gender stratum, along with post-stratified estimates applied to the age and gender distributions of study participants and eligible residents for whom demographic data were available.

Group	Adjusted seroprevalence (95% CI)
<18, Female	57.49% (52.37-62.73%)
<18, Male	50.91% (45.59-56.33%)
18-29, Female	52.73% (47.00-58.78%)
18-29, Male	46.11% (40.10-52.50%)
30-44, Female	47.44% (42.00-53.08%)
30-44, Male	40.89% (35.17-46.95%)
45-59, Female	42.25% (36.34-48.68%)
45-59, Male	35.91% (29.80-42.46%)
≥60, Female	47.83% (39.68-56.35%)
≥60, Male	41.30% (33.18-49.79%)
Study participants	
All	47.93% (44.22-52.10%)
Female	50.29% (46.32-54.82%)
Male	44.62% (40.13-49.44%)
Children	54.38% (49.57-59.29%)
Adults	45.37% (41.49-49.72%)
Eligible residents	
All	47.70% (43.99-51.83%)
Female	50.37% (46.42-54.89%)
Male	44.25% (39.77-49.07%)
Children	54.44% (49.63-59.35%)
Adults	45.10% (41.23-49.45%)

Household composition and increased risk among women
In addition to differences in employment and income, there were differences between adult women and men in their household composition. Compared to men, women more often lived in larger households and with children. Moreover, women were more likely than men to be the single adult in a household with children (Fig C, panel A). Women with larger households dependent on a single income were thus more likely to experience risk from both physical crowding and poverty.

Figure C: Household composition. A: Household composition by gender. Women were more likely to be the sole adult in households with multiple children. B: Variation in seroprevalence among adults by household composition (number of adults and presence of children). Adults living with children were more likely to be seropositive. Accounting for household composition, there was no statistically significant difference in seroprevalence between men and women.

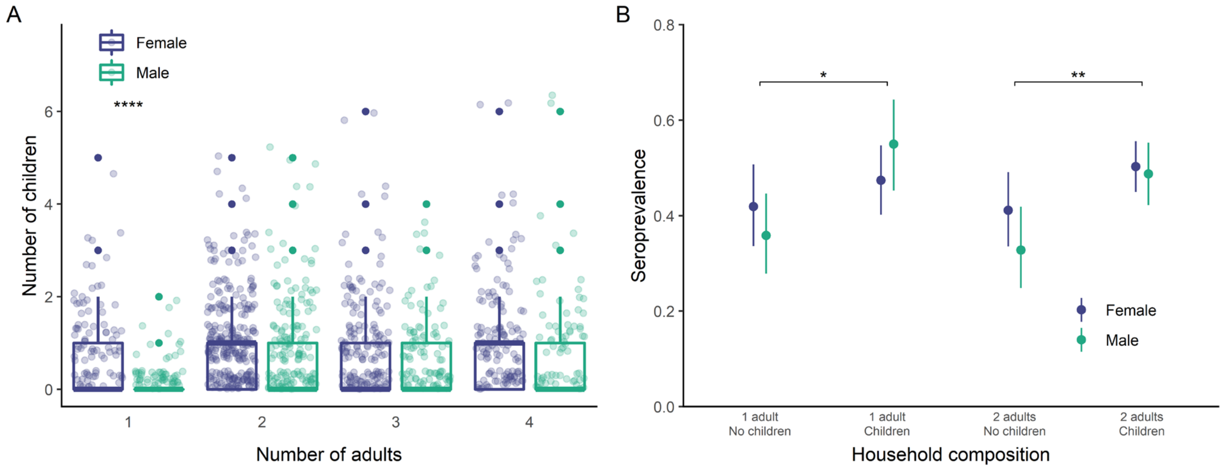

Table D: Univariable logistic regression analyses of individual and household characteristics associated with seropositivity

Variable	Category	OR (95% CI)
Age	<18	1.71 (1.35-2.17)
	18-29	1.24 (0.96-1.60)
	30-44	(Ref)
	45-59	0.82 (0.62-1.08)
	≥60	0.97 (0.68-1.38)
Gender	Male	(Ref)
	Female	1.21 (1.02-1.45)
Race	Black	(Ref)
	Brown	1.17 (0.97-1.40)
	Other	1.30 (0.68-2.48)
	White	1.17 (0.77-1.76)
Marriage or stable union	No	(Ref)
	Yes	0.74 (0.60-0.90)
Years of schooling	0-6	1.05 (0.85-1.28)
	7-9	1.30 (1.03-1.66)
	>9	(Ref)
Employment	Formal	(Ref)
	Informal	0.88 (0.62-1.24)
	Unemployed	1.15 (0.85-1.56)
Daily per capita income (USD)	<1.25	1.31 (1.01-1.71)
	1.25-2.49	0.91 (0.65-1.28)
	2.5-4.99	0.79 (0.56-1.13)
	≥5	(Ref)
Total residents in household	Per person	1.17 (1.12-1.23)

Table E: Adherence to non-pharmaceutical interventions

Variable (N)	Frequency (per week)	Number (%)	SARS-CoV-2 IgG +	
			N	Proportion (95% CI)
**Handwashing**	Never	136 (6.7%)	64	47.1% (38.5-55.8%)
	Some days	466 (22.9%)	233	50.0% (45.5-54.5%)
	Most days	309 (15.2%)	162	52.4% (46.7-58.1%)
	Every day	1127 (55.3%)	521	46.2% (43.3-49.2%)
	Total	*2038*		
**Alcohol-based sanitizer use**	Never	174 (8.5%)	74	42.5% (35.1-50.2%)
	Some days	499 (24.5%)	248	49.7% (45.2-54.2%)
	Most days	303 (14.9%)	158	52.1% (46.4-57.9%)
	Every day	1062 (52.1%)	500	47.1% (44.0-50.1%)
	Total	*2038*		
**Mask use**	Never	86 (4.2%)	36	41.9% (31.5-53.0%)
	Some days	474 (23.4%)	241	50.8% (46.2-55.4%)
	Most days	309 (15.2%)	154	49.8% (44.1-55.5%)
	Every day	1159 (57.1%)	544	46.9% (44.0-49.9%)
	Total	*2028*		
**Physical distancing >2m**	Never	384 (18.9%)	187	48.7% (43.6-53.8%)
	Some days	702 (34.5%)	348	49.6% (45.8-53.3%)
	Most days	385 (18.9%)	190	49.4% (44.3-54.5%)
	Every day	564 (27.7%)	254	45.0% (40.9-49.3%)
	Total	*2035*		
**Isolation**	Never	427 (21.0%)	205	48.0% (43.2-52.9%)
	Some days	726 (35.6%)	366	50.4% (46.7-54.1%)
	Most days	390 (19.1%)	176	45.1% (40.1-50.2%)
	Every day	494 (24.3%)	232	47.0% (42.5-51.5%)
	Total	*2037*		

Sensitivity analyses
A sensitivity analysis using a normalized OD cutoff of 1.1 (vs. 0.8 in the main analysis) did not significantly affect our findings (Table F). Similarly, using different age categories that distinguish children <13 years of age from adolescents aged 13-17 years did not significantly change our findings (Table G).

Table F: Sensitivity analysis with higher seropositivity threshold. Statistically significant effect sizes are indicated in bold.

Variable	Category	OR (95% CI)		
		Model 1	*Model 2*	*Model 3*
**Age**	<18	**2.46 (1.68-3.61)**		
	18-29	**1.65 (1.13-2.42)**	**1.68 (1.21-2.35)**	**1.58 (1.14-2.20)**
	30-44	(Ref)	(Ref)	(Ref)
	45-59	0.87 (0.57-1.34)	0.95 (0.65-1.38)	0.95 (0.65-1.37)
	≥60	1.05 (0.59-1.88)	1.04 (0.63-1.71)	1.06 (0.65-1.74)
**Gender**	Male	(Ref)	(Ref)	(Ref)
	Female	**1.61 (1.22-2.12)**	**1.66 (1.23-2.22)**	**1.63 (1.22-2.19)**
**Daily per capita income (USD)**		1.03 (0.99-1.08)	1.03 (0.99-1.07)	1.03 (0.99-1.08)
**Children in home (Y/N)**			**1.36 (1.01-1.82)**	
**Total residents**		**1.14 (1.04-1.24)**		**1.14 (1.05-1.23)**
AIC		*1985*	*1447*	*1442*
ICC		*0.32*	*0.133*	*0.125*

Table G: Sensitivity analysis with different age categories. Statistically significant effect sizes are indicated in bold.

Variable	Category	OR (95% CI)
**Age**	<13	**1.85 (1.20-2.86)**
	13-17	**2.37 (1.48-3.78)**
	18-29	**1.56 (1.07-2.29)**
	30-44	(Ref)
	45-59	0.88 (0.58-1.35)
	≥60	0.95 (0.53-1.70)
**Gender**	Male	(Ref)
	Female	**1.49 (1.13-1.96)**
**Daily per capita income (USD)**		1.03 (0.98-1.07)
**Total residents**		**1.14 (1.04-1.25)**
AIC		*2022*
ICC		*0.326*

SARS-CoV-2 serology results were available for all residents of 431 households, accounting for 979 individuals (48.0% of our total study population). In order to assess the robustness of our findings, we compared the characteristics of households with and without complete serological data for all residents. Panels A and B in Fig D show the distribution of total number of residents and number of seropositive individuals per household. Odds ratios in univariable analyses did not differ significantly between the whole study population and the subset of households with complete serological data (Fig D, panel C). We repeated our primary analyses, using only data from households with complete serological data. The distribution of age and association with SARS-CoV-2 seropositivity was similar among the subset of households with complete serological data (Fig E). The associations between gender, household composition, income, and SARS-CoV-2 seropositivity remained similar in analyses including only households with complete serological data (Fig F).

Figure D: Comparison of households with complete serological data and overall study population. Distribution of total number of residents (A) and seropositive individuals (B) in each household. Panel C shows univariable odds ratios for all households compared to those with serological data for all residents ("complete").

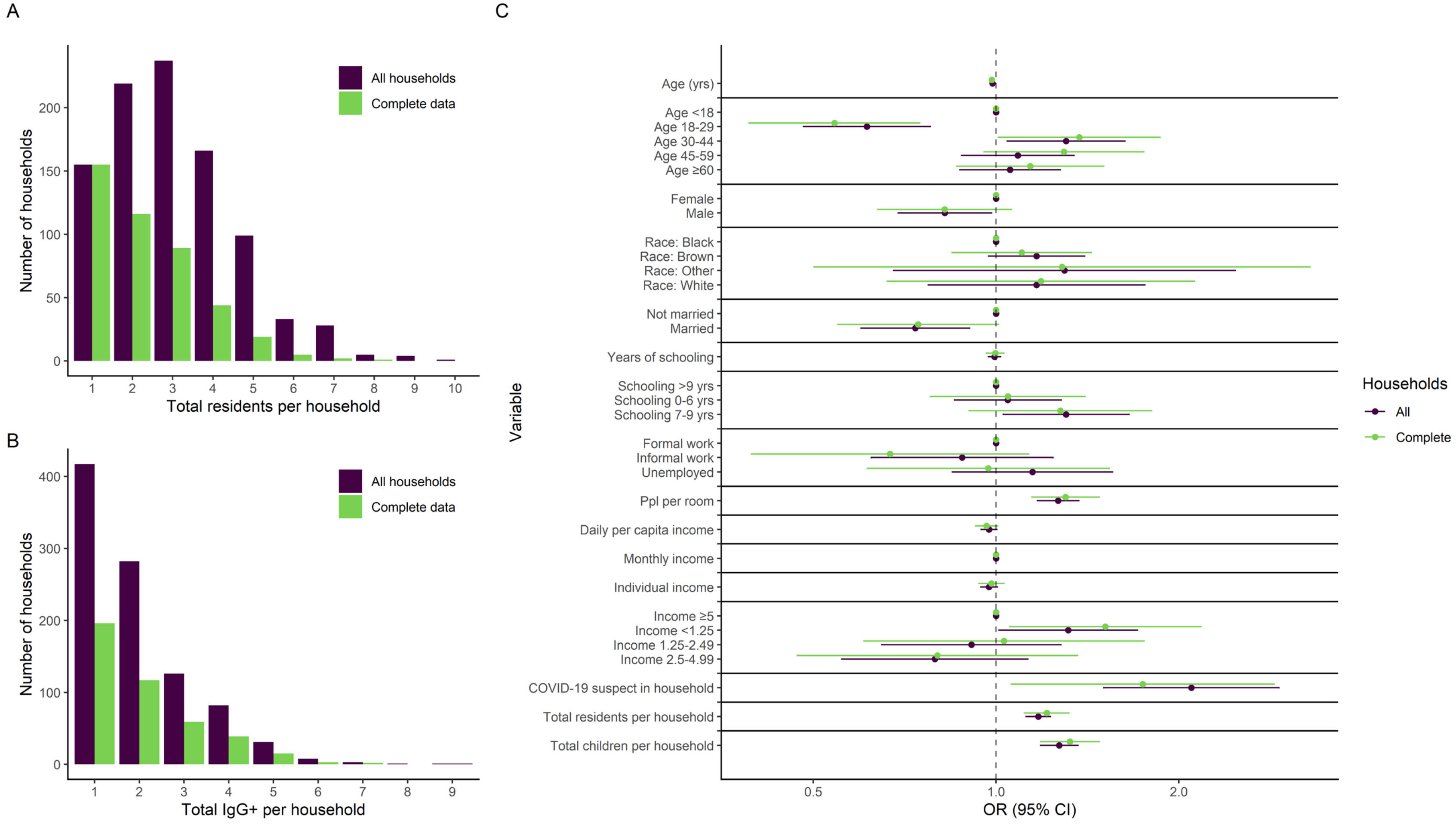

Figure E: Sensitivity analysis on effect of age among households with complete serological data. A: Distribution of age between SARS-CoV-2 seronegative and seropositive individuals. B: Relationship between age and SARS-CoV-2 seropositivity by gender, as estimated using a generalized additive model. C: Seroprevalence by age, with and without presence of children in the household. D: Comparison of household size and age between SARS-CoV-2 seronegative and seropositive individuals. Asterisks indicate statistically significant differences (two-tailed t-test, Bonferroni-adjusted p<0.05: *; <0.01: **; <0.001: ***; α= 0.0125, 0.01 in panels C-D).

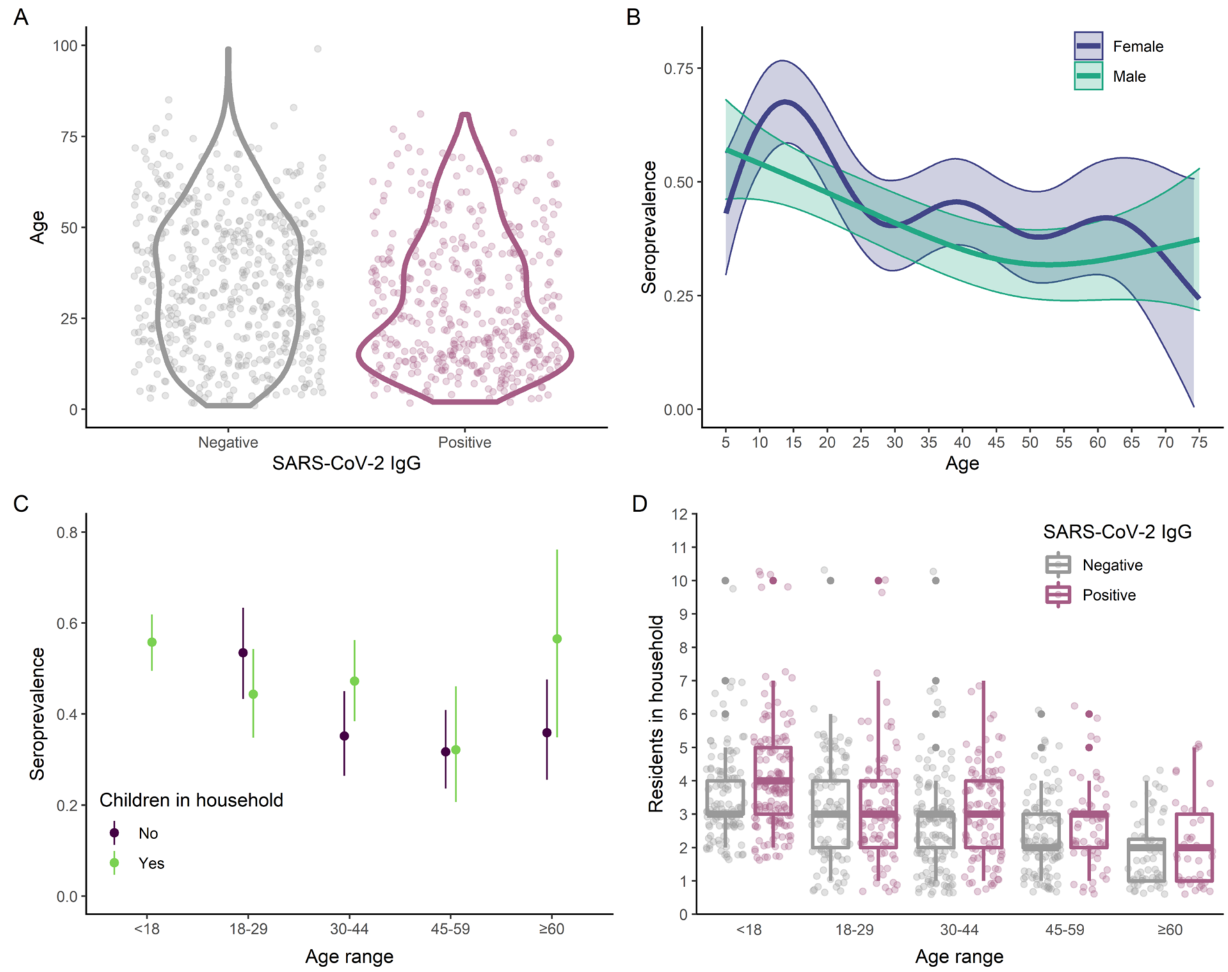

Figure F: Sensitivity analysis on the effects of gender, income, and household composition, among households with complete serological data. A: Seroprevalence by daily per capita income, employment and gender. B: Comparison of household composition, assessed by number of adults and children, by gender. C: Variation in seroprevalence by household composition. Asterisks indicate statistically significant differences (two-tailed t-test, Bonferroni-adjusted p<0.05: *; <0.01: **; <0.001: ***; α= 0.0125, 0.0125, 0.025 in panels A-C).

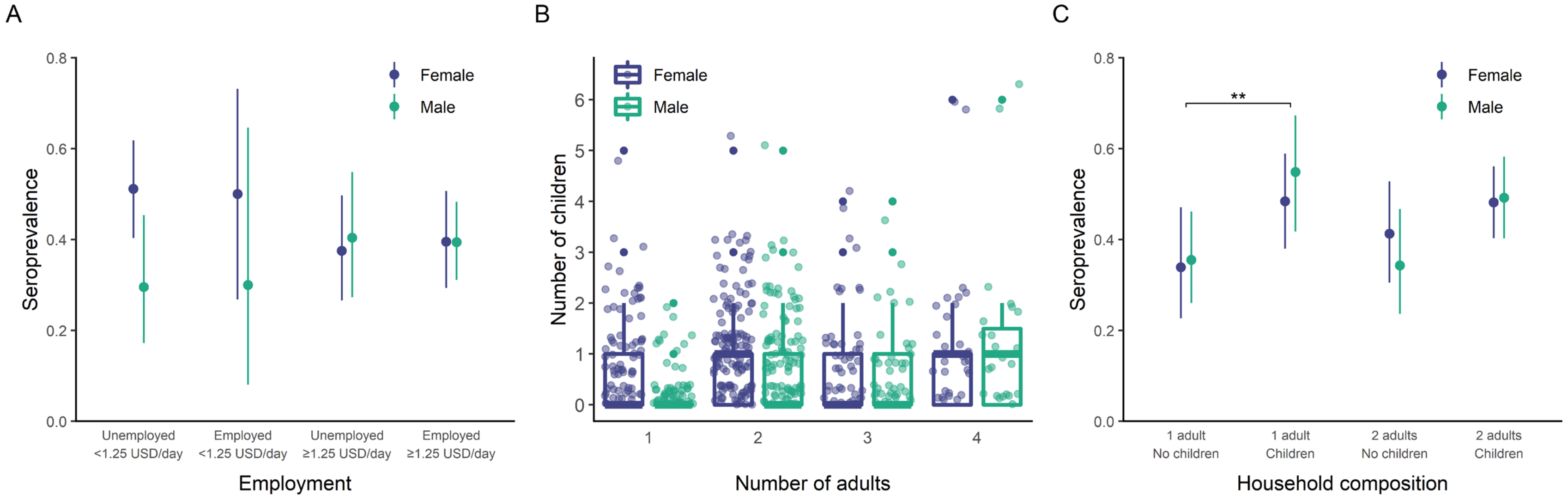

In order to assess the robustness of our findings to missing data, we used the R "mice" package [6] to impute all the independent variables included in the univariable analyses. We assessed model convergence visually and with the Rhat statistic. We generated 5 imputed datasets and conducted a multivariable logistic regression with each imputed dataset, including all the variables evaluated in univariable models. We then computed pooled estimates of the odds ratios and 95% confidence intervals (Table H).

Table H: Multivariable analyses with multiple imputation. Statistically significant effect sizes are indicated in bold.

Variable	Category	OR (95% CI)	
		Model 1	*Model 2*
Age	<18	**1.72 (1.15-2.57)**	**1.71 (1.15-2.54)**
	18-29	1.39 (0.93-2.06)	1.30 (0.88-1.94)
	30-44	(Ref)	(Ref)
	45-59	0.75 (0.48-1.16)	0.75 (0.49-1.15)
	≥60	1.02 (0.56-1.86)	1.05 (0.58-1.90)
Daily per capita income (USD)		1.02 (0.97-1.08)	1.03 (0.98-1.09)
Race	Black	(Ref)	(Ref)
	Brown	1.26 (0.94-1.69)	1.27 (0.95-1.70)
	Other	1.42 (0.52-3.83)	1.45 (0.54-3.91)
	White	1.14 (0.61-2.13)	1.12 (0.60-2.08)
Marriage or stable union	No	(Ref)	(Ref)
	Yes	0.81 (0.58-1.14)	0.79 (0.57-1.10)
Employment	Unspecified employment	(Ref)	(Ref)
	Formal employment	1.13 (0.53-2.40)	1.03 (0.49-2.17)
	Informal employment	0.86 (0.44-1.69)	0.80 (0.41-1.55)
	Unemployed	1.09 (0.63-1.90)	1.04 (0.60-1.80)
Years of schooling	0-6 years	1.00 (0.69-1.45)	0.99 (0.69-1.43)
	7-9 years	1.17 (0.79-1.71)	1.15 (0.78-1.68)
	>9 years	(Ref)	(Ref)
Gender	Male	(Ref)	(Ref)
	Female	**1.52 (1.16-2.00)**	**1.52 (1.16-1.99)**
Presence of children	No	(Ref)	(Ref)
	Yes	**1.54 (1.04-2.27)**	
Total residents in household			**1.21 (1.09-1.34)**
Mean AIC		*2602*	*2593*

References

1. Jääskeläinen AJ, Kekäläinen E, Kallio-Kokko H, Mannonen L, Kortela E, Vapalahti O, et al. Evaluation of commercial and automated SARS-CoV-2 IgG and IgA ELISAs using coronavirus disease (COVID-19) patient samples. Eurosurveillance. 2020;25. doi:10.2807/1560-7917.ES.2020.25.18.2000603.

2. Manthei DM, Whalen JF, Schroeder LF, Sinay AM, Li S-H, Valdez R, et al. Differences in Performance Characteristics Among Four High-Throughput Assays for the Detection of Antibodies Against SARS-CoV-2 Using a Common Set of Patient Samples. American Journal of Clinical Pathology. 2021;155: 267–279. doi:10.1093/ajcp/aqaa200.

3. Hitchings MDT, Cummings DAT, Grais RF, Isanaka S. A mixture model to assess the the immunogenicity of an oral rotavirus vaccine among healthy infants in Niger. Vaccine. 2020;38: 8161–8166. doi:10.1016/j.vaccine.2020.10.079.

4. Hitchings MDT, Patel EU, Khan R, Srikrishnan AK, Anderson M, Kumar KS, et al. A mixture model to estimate SARS-CoV-2 seroprevalence in Chennai, India. Epidemiology; 2022. doi:10.1101/2022.02.24.22271002.

5. Stringhini S, Wisniak A, Piumatti G, Azman AS, Lauer SA, Baysson H, et al. Seroprevalence of anti-SARS-CoV-2 IgG antibodies in Geneva, Switzerland (SEROCoV-POP): a population-based study. The Lancet. 2020;396: 313–319. doi:10.1016/S0140-6736(20)31304-0.

6. Buuren S van, Groothuis-Oudshoorn K. mice: Multivariate Imputation by Chained Equations in R. J Stat Soft. 2011;45. doi:10.18637/jss.v045.i03.
